# Effects of Marigold Extract and Carophyll Red on Growth, Body Color Development, Antioxidant Properties, and Innate Immunity in the Ornamental Fish Golden Severum (*Heros efasciatus*)

**DOI:** 10.3390/life14121660

**Published:** 2024-12-13

**Authors:** Jung-Jin Park, Jun-Sung Bae, Chae-Won Lee, Chan-Young Yang, Eun-Ha Jeong, Kwan-Ha Park, Jung-Soo Seo, Mun-Gyeong Kwon, Ji-Hoon Lee

**Affiliations:** 1 Department of Aquatic Life Medicine, Kunsan National University, Gunsan 54150, Republic of Korea; pjj3770@gmail.com (J.-J.P.); 1301806@kunsan.ac.kr (J.-S.B.); didqocn2007@naver.com (C.-W.L.); misha9703@kunsan.ac.kr (C.-Y.Y.); dmsgk1532@kunsan.ac.kr (E.-H.J.); khpark@kunsan.ac.kr (K.-H.P.); 2 Aquatic Disease Control Division, National Fisheries Products Quality Management Service, 337 Haeyang-ro, Yeongdo-gu, Busan 49111, Republic of Korea; jsseosoo@korea.kr (J.-S.S.); mgkwon@korea.kr (M.-G.K.)

**Keywords:** antioxidant activity, body color, carophyll red (CR), innate immunity, marigold extract (MG)

## Abstract

The body color state is an important determinant of the value of golden severum (*Heros efasciatus*)—a popular ornamental fish. The use of dietary supplements to improve the color development and health of this species is unexplored. Herein, the effects of marigold extract (MG) and carophyll red (CR) are examined on the growth, body color development, antioxidant properties, and innate immunity in golden severum. Fish were maintained under controlled water quality conditions (pH, temperature, and dissolved oxygen) and fed six experimental diets containing either 0% MG and CR, 1% MG, 2% MG, 5% MG, 0.5% CR, or 2% CR for five weeks. Both MG and CR significantly decreased lipid peroxide levels in hepatic tissues. In contrast, only MG enhanced the activities of reactive oxygen species (ROS)-scavenging enzymes (superoxide dismutase and catalase). Although MG and CR decreased the respiratory burst activity of splenic leukocytes, other innate immune parameters remained unchanged. Additionally, MG and CR stimulated body color development patterns in golden severum that reflect their unique coloring principles. The ROS-scavenging abilities of MG and CR appear to be related to their antioxidant activity. Hence, MG and CR at the optimal levels of 1.0% and 0.5%, respectively, can improve the body color of golden severum and protect against oxidative stress.

## 1. Introduction

Over 700 fish species are raised worldwide for ornamental purposes [1], the value of which is estimated using parameters such as size, body shape, and body color. Body color is considered one of the most important characteristics influencing the preferences of aquarists [2]. A non-invasive method to improve the color characteristics of ornamental fish is to feed them non-toxic colorants through their diet. These colorants, such as carotenoids, are absorbed and deposited in the skin or muscle tissues of fish, where they interact with chromatophores—specialized pigment-containing cells [3]. For this purpose, natural substances, such as carotenoids, melanin, flavin, and purines, have been tested [4]. Many such natural substances also impart beneficial pharmacological effects. For instance, numerous carotenoids improve animal body color while acting as immune system activators and antioxidants [5,6].

Marigold plants are an important source of carotenoids. Although there are numerous marigold plant variants worldwide, the two major cultivars used for commercial purposes are the common marigold, *Calendula officinalis*, native to Asia, Europe, Macaronesia, and Mediterranean regions, and the Mexican marigold, *Tagetes erecta*, of South American origin [7]. *Tagetes erecta* has long been cultivated for ornamental and medicinal purposes worldwide [8]. Its flower is primarily utilized as a diuretic, diaphoretic, and hypotensive agent in Asian countries. The extract contains high levels of lutein, a xanthophyll carotenoid commonly used to delay the macular degeneration of the retina [9]. The extract is also utilized in animal feed due to its antioxidant and color-improving effects, which enhance consumer preference [10,11,12].

Canthaxanthin (CTX) is a synthetic xanthophyll carotenoid developed to impart optimum coloring effects in broiler skin and egg yolk [13]. Carophyll red (CR), a commercially available CTX, is bright red in color and highly water-soluble. It has been marketed by the Swiss company DSM (Basel, Switzerland), claiming effects like growth stimulation, antioxidant activity, and egg quality improvement [14].

Previous studies have demonstrated that MG and CR can enhance pigmentation in food fish, such as rainbow trout (*Oncorhynchus mykiss*), by depositing carotenoids in the skin and muscle tissues. These colorants have primarily been used to improve coloration in food fish and poultry, with limited applications in ornamental fish.

Golden severum (*Heros efasciatus*), a cichlid fish inhabiting Latin American countries, develops a yellowish color upon maturation, and males develop peculiar red spots during spawning seasons [15]. Due to its elegant body shape and color, golden severum is popular, representing a major commodity for national and international trade. The body color state is an important factor that determines the value of this ornamental fish. Although maintaining the health of the fish is also highly desirable, the use of dietary supplements to improve color development and the overall health of golden severum has not been explored.

This study delineates the effects of two coloring substances, lutein-containing marigold extract (MG) and commercially produced CTX-containing CR, on the general growth, body color development, innate immunity, and antioxidant activities of golden severum.

## 2. Materials and Methods

### 2.1. Experimental Reagents

Histopaque-1077, nitrotetrazolium blue chloride, *Micrococcus lysodeikticus* ATCC 4698, phorbol 12-myristate 13-acetate (PMA), and zymosan A were obtained from Sigma (St. Louis, MO, USA). In addition, 2-Phenoxyethanol was purchased from Junsei (Tokyo, Japan) to be used as an anesthetizing agent during color measurement and blood sample collection. Reagents were dissolved in phosphate-buffered saline (PBS; Corning, New York, NY, USA) to measure respiratory burst activity in leukocytes.

### 2.2. Golden Severum Husbandry

Golden severum (*H. efasciatus*) (9.22 ± 0.61 g) were obtained from a local ornamental fish dealer. The fish were maintained for four weeks in 150 L recirculation-type glass aquaria (90 × 45 × 45 cm, l × b × h) with 14:10 h light/dark cycles at 26 ± 0.5 °C to allow acclimatization to the laboratory conditions and the maximum disappearance of pre-existing body colors, which may have developed due to other coloring constituents. The dissolved oxygen level was maintained at >9 mg/L using an electrical aerator. During the acclimation period, water quality parameters were regularly monitored to ensure optimal conditions for the fish. Temperature, pH, and dissolved oxygen were measured daily using a YSI 556 multiparameter instrument (YSI, Yellow Springs, OH, USA), while nitrate, nitrite, ammonia, and total hardness were measured weekly using HI test kits (HANNA instruments, Seoul, Korea). The ranges observed during the experiment were as follows: pH, 6.7 ± 0.3; nitrate, 3 ± 1 mg/L; nitrite, 0.1 ± 0.05 mg/L; ammonia, 1 ± 0.5 mg/L; total hardness, 80–120 mg/L CaCO_3_). These levels were consistently maintained within optimal ranges based on the aquaculture standards for cichlid fish [15]. The conditions in the aquaria during the experiment were the same as during the acclimation period. A total of 20 fish were maintained in each aquarium, and all experimental groups comprised triplicate aquaria. During acclimation, a commercial diet (Start No. 2, Chunhajeil Feed, Gyeongnam, Korea) was provided daily at 10:00 a.m., at a rate of 1.0% of body weight. This commercial diet is the same as the feed used to adsorb the two test substances (MG or CR). Growth and color development were assessed in all fish. For all other parameters, a portion of the animals were randomly selected to ensure data reliability due to differences in sampling times for individual fish. All fish experiments were conducted following the guidelines of the Institutional Animal Care and Use Committee (IACUC, No. 2018) of the Kunsan National University, Korea.

### 2.3. Preparation of Experimental Diets

The two test substances, MG (prepared from Mexican marigold *T. erecta* flower) and CTX-containing CR, were obtained from Honson Ingredients (Toronto, ON, Canada) and DSM (Basel, Switzerland), respectively. The lutein content in MG provided by the manufacturer was 5% (Honson Ingredients, https://www.honsons.com), while that of CTX in CR was 10% (DSM, https://www.dsm.com). MG was adsorbed onto the commercial feed at 1%, 2%, and 5% after dissolving in 50% ethanol. Subsequently, the feed was air-dried at room temperature, resulting in a moisture content of 4%. CR was similarly adsorbed onto the feed at 0.5% and 2%. Other nutrient contents were not adjusted despite slight changes due to the inclusion of the test substances. A one-week feed supply of the prepared diets was stored at 4 °C in sealed containers. To prevent the leaching of water-soluble ingredients before intake, the feed was coated with fish oil (Sigma, St. Louis, MO, USA) immediately before administration. The control feed was similarly coated. Analysis of the control diet [16] revealed the following composition (in percentage dry matter): crude protein, 54.8; crude fat, 6.2; crude ash, 16.7; and moisture, 19.4. The provider confirmed that no carotenoids (either MG or CR) were present in the control diet. Experimental diets were fed to satiation levels once daily at 10:00 a.m. for five weeks by retrieving uneaten feed 10 min after the supply to estimate actual consumption. The moisture content was ignored when calculating the feed consumption.

### 2.4. Growth and Color Development Assessments

After five weeks of feeding, fish were anesthetized with 2-phenoxyethanol (500 μL/L) and counted to determine the survival rate of each group. To evaluate the effects of MG and CR on growth, body weights were measured to determine weight gain (WG) and specific growth rate (SGR), calculated using Equations (1) and (2) as follows:

WG (%) = 100 × Final weight (g) − Initial weight (g)/Initial weight (g)(1)

SGR (%) = 100 × log *e* final mean weight − log *e* initial mean weight/Experimental days (2)

**Figure 1:**
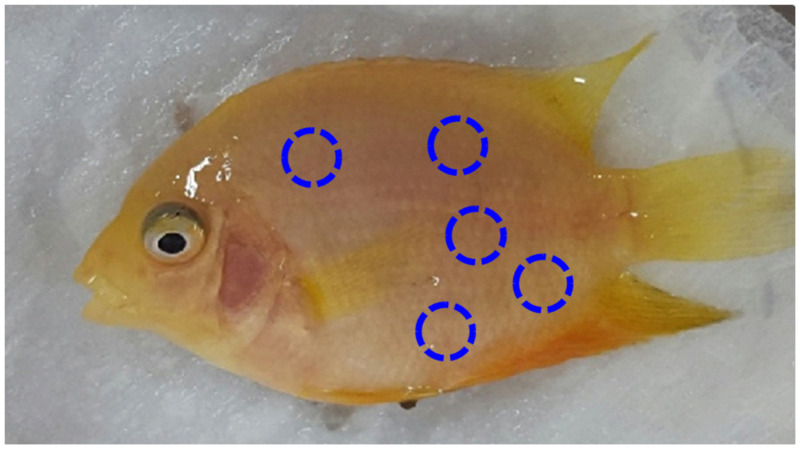
Approximate measurement points of chromaticity (body color) on a typical golden severum fed a 1.0% MG diet for five weeks.

To estimate body color, fish were mounted on a chromatometer (CM-5; Minolta, Tokyo, Japan) under light anesthesia [17]. Values were expressed using Hunter chromaticity scales [18] in terms of lightness (L), red/green chromaticity (a), and yellow/blue chromaticity (b). The background reference values of the standard mounting board were L = 101.42, a = 0.08, and b = −0.32. Figure 1 depicts five approximate points at which the color values were taken to obtain the mean values.

### 2.5. Blood and Tissue Sampling and Processing

After evaluating growth based on the bulk mass of fish, the chromaticity of all individual fish was measured. For the measurement of other parameters, four fish from each of the three tanks per group were randomly selected to obtain blood samples by severing the tail end. The liver and spleen were aseptically isolated from the fish after sacrifice.

The collected blood was clotted, and serum was separated via centrifugation (Combi-514R; Hanil Scientific, Incheon, Korea) at 3000× *g* for 30 min at 4 °C. Serum was used for lysozyme activity measurements, and hepatic tissues were homogenized for biochemical anti-oxidative assays in five volumes of Tris buffer (50 mM Tris (pH 7.5), 1 mM EDTA, 1 mM DL-dithiothreitol, and 150 mM NaCl). The homogenate was centrifuged for 20 min at 12,000× *g* and 4 °C to obtain the supernatant [19]. The hepatic supernatant was used to determine superoxide dismutase (SOD), glutathione-S-transferase (GST), and catalase (CAT) activities, as well as thiobarbituric acid reactive substance (TBARS) levels. The spleen was homogenized for 30 min at 2500× *g* in five volumes of Dulbecco’s modified Eagle’s medium (DMEM; Gibco, New York, NY, USA) and homogenized after adding 3 mL of Histopaque-1077. The spleen buffy-coat layer was retrieved to isolate leukocytes. Leukocytes were counted using a hemocytometer (DHC-N01-5; Cheonan, Korea) and adjusted to 1.25 × 10^7^ cells/mL in DMEM. This leukocyte suspension was used to measure reactive oxygen species (ROS) levels and phagocytic activity. Serum used for lysozyme activity was stored at −80 °C until analysis; the other tissue samples (hepatic and splenic tissues) were used for analysis immediately after collection.

### 2.6. Antioxidant Biochemical Parameters

SOD activity was measured as described previously [20], using a commercial assay kit (SOD assay kit-WST; Dojindo, Kumamoto, Japan). In principle, 2-(4-iodophenyl)-3-(4-nitrophenyl)-5-2(2,4-disulfophenyl)-2H-tetrazolium monosodium salt or water-soluble tetrazolium salt-1 (WST-1) was converted to WST-1 formazan by superoxide anions (O_2_^−^) generated via the xanthine/xanthine oxidase reaction. SOD activity inhibited the conversion process. The reaction mixture containing the liver supernatant (with SOD) was incubated for 20 min at 37 °C, and absorbance was monitored at 450 nm using a microplate reader (EL808l; BioTek, Winooski, VT, USA).

GST activity was measured using a commercial kit (GST Assay kit; Cayman Chemical, Ann Arbor, MI, USA) according to a previously described protocol [21]. The kit was used to estimate the rate of conjugate formation between glutathione and 1-chloro-2,4-dinitrobenzene (CDNB), when liver homogenates (which contain GST) were incubated with glutathione and CDNB. The reaction was continued for 10 min at 25 °C, followed by absorbance measurement at 340 nm using a microplate reader.

CAT activity was assessed using a commercial kit (CAT Assay kit; Cayman Chemical); formaldehyde formation was estimated based on the reaction of hepatic tissue CAT with methanol. Formaldehyde formation was estimated by spectrophotometrically monitoring the formation of a conjugate between 4-amino-3-hydrazino-5-mercapto-1,2,4-triazole and formaldehyde [22]. The final conjugate product was determined using a microplate reader at a wavelength of 540 nm.

TBARS levels were measured using the malondialdehyde (MDA) method [23]. The reaction product was measured spectrophotometrically at 530 nm using a microplate reader. The reactions were assayed using a commercial kit (TBARS Assay kit, Cayman Chemical).

### 2.7. Innate Immunity Parameters

Serum lysozyme activity was estimated according to a previously described method [24], with slight modifications. Briefly, 30 μL of severum serum was mixed in a 96-well microplate with 70 μL of 2 mg/mL freeze-dried *Micrococcus lysodeikticus* cell suspension in PBS (pH 5.8). The change in absorbance of the mixture was kinetically monitored for 4 min at 405 nm after incubation at 25 °C. The enzyme activity was defined as one unit per 0.001 absorbance unit decrease per minute.

The splenic leukocytes used in these assays were prepared as described in Section 2.5. The ROS production by splenic leukocytes was measured as previously described [25]. The prepared leukocytes (200 μL) were loaded into a 96-well microplate and centrifuged at 120× *g* for 5 min at room temperature to collect the leukocytes. The supernatant was collected and rinsed twice with PBS (pH 7.4). Subsequently, 100 μL of PBS containing PMA (1 μg/mL) and nitroblue tetrazolium (1 mg/mL) was added to the cells and incubated in the dark for 1 h at 25 °C. After incubation, leukocytes were rinsed twice with PBS; 100 μL of 70% methanol was added, and the cells were washed twice with PBS. The cells were lysed with 120 μL KOH (0.2 M) and 140 μL dimethyl sulfoxide. The absorbance of the cell lysates was measured using a microplate reader at 620 nm.

To measure the phagocytic activity of splenic leukocytes [26,27], 1.25 × 10^7^ cells/mL were added to a Nunc Lab-Teck II chamber slide and pre-incubated in the dark for 12 h at 25 °C. Next, 10 μL of zymosan (1 × 10^6^ cell/mL) was added; the slide was incubated at 25 °C for 1 h and centrifuged for 5 min at 120× *g* and room temperature to remove the supernatant. Adherent leukocytes were rinsed twice with PBS (pH 7.4), and then, 200 μL of 70% methanol was added. The chamber was air-dried and stained with Diff-Quik dye (Sysmex, Kobe, Japan). After drying, 100 leucocytes were randomly selected and examined using a light microscope to enumerate zymosan-engulfed leukocytes (phagocytosis rate, PR) and the total number of zymosan particles in a cell (phagocytosis index, PI).

### 2.8. Statistical Analysis

Experimental results were expressed as the mean ± standard deviation (SD). Statistical analysis of each parameter was performed as follows: general growth, *n* = 3 (tank mean: 20 fish per tank); body color values, *n* = 60 (triplicate tanks of 20 fish each); antioxidant and innate immune parameters, *n* = 12 (4 fish from each of the three tanks). To determine significant differences between the control and different diet-fed groups, a one-way analysis of variance (ANOVA) followed by Dunnett’s multiple comparison test was performed. Differences were considered statistically significant at *p* < 0.05.

## 3. Results

### 3.1. General Growth Parameters

During the 5-week feeding period, general growth parameters, expressed as WG and SGR, showed no significant differences between the control and any of the experimental groups (Table 1). These results indicate that the inclusion of MG or CR in the diet did not have a detrimental or beneficial effect on the growth of golden severum within the tested concentration ranges.

### 3.2. Effects of MG and CR on Body Color Development

**Figure 2:**
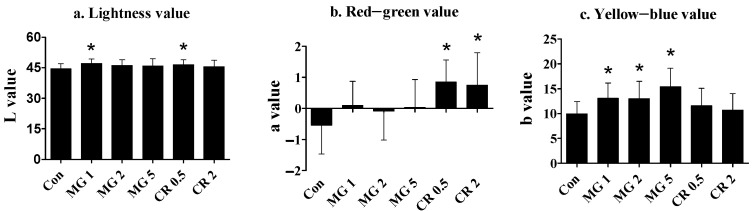
Effects of 5-week MG and CR feeding on body color values of golden severum. Data are expressed as the mean ± standard deviation from 60 fish (triplicate tanks of 20 fish each). * Significant difference from control at *p* < 0.05 with Dunnett’s analysis. Con represents the control group, and the numbers on the *X*-axis indicate the diet percentage. (**a**) Lightness value (L), representing the brightness of the body color, measured using a chromatometer; (**b**) Red–green value (a), indicating the red or green intensity of the body color; (**c**) Yellow–blue value (b), reflecting the yellow or blue intensity of the body color. Values were expressed using Hunter chromaticity scales, with background reference values for the mounting board set to L = 101.42, a = 0.08, and b = −0.32.

The activity of the test substances on body color development was estimated using Hunter chromaticity indices. Figure 2 illustrates the effects of MG and CR on body color after a 5-week feeding period. Compared with the control, there was a slight increase in the lightness value (L value, Figure 2a) in the lower test concentration groups of MG (1%) and CR (0.5%). Feeding with higher concentrations of both substances led to insignificant changes in lightness. Regarding the red–green chromaticity level (value a, Figure 2b), CR-fed fish exhibited higher chromaticity in the groups with 0.5% and 2% CR. In contrast, MG did not influence this value at concentrations of 1.0–5.0%. The yellow–blue chromaticity value (value b, Figure 2c) was significantly enhanced by feeding MG at all concentrations (1.0–5.0%); CR did not influence the yellow–blue value at concentrations up to 2.0%.

### 3.3. Antioxidant Parameters

**Figure 3:**
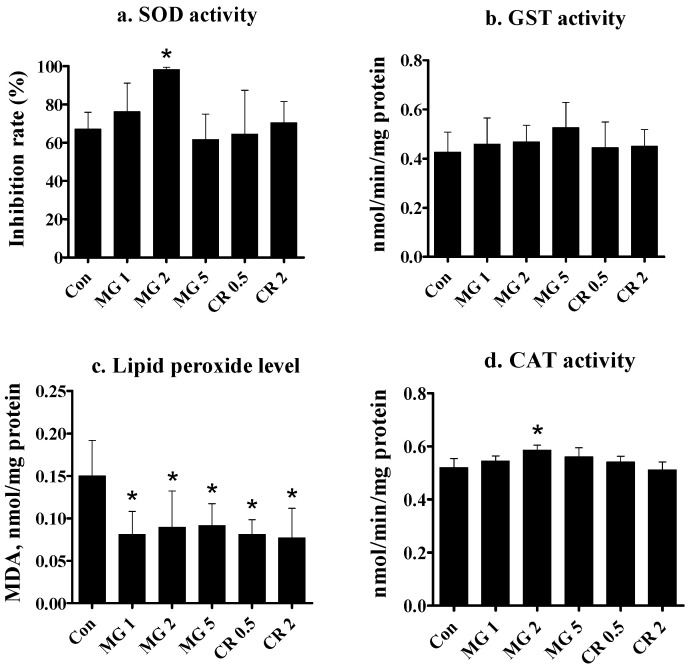
Effects of 5-week MG and CR feeding on hepatic antioxidant parameters of golden severum. Data are expressed as the mean ± standard deviation from 12 fish per group (4 fish from each of the 3 tanks). * Significant difference from control at *p* < 0.05 with Dunnett’s analysis. Con, control; numbers on the *X*-axis denote diet percentage. (**a**) SOD activity (inhibition rate, %), reflecting the superoxide dismutase-mediated inhibition of WST-1 formazan formation; (**b**) GST activity (nmol/min/mg protein), based on glutathione conjugation with CDNB; (**c**) Lipid peroxide level (MDA, nmol/mg protein), indicating malondialdehyde levels; (**d**) CAT activity (nmol/min/mg protein), measured via formaldehyde formation.

Biochemical parameters were examined after the feeding period to estimate the anti-oxidative activities of the test substances. Assessments were performed for liver tissues (Figure 3). SOD activity was elevated after supplementation with MG in a biphasic manner; the 1.0–2.0% groups exhibited an increasing tendency, whereas the 5% group showed an activity level similar to that of the control. In contrast to MG, CR at 0.5 or 2.0% did not induce changes in SOD activity (Figure 3a).

The two test substances did not influence hepatic GST enzyme activity after the 5-week feeding period (Figure 3b). A significant reduction in TABRS, that is, lowered lipid peroxide (MDA) concentration, was observed in all groups fed MG or CR (Figure 3c). Hepatic CAT activity increased after feeding with 2% MG. The hepatic CAT activity of the 1% and 5% MG groups did not differ from the control group (Figure 3d). In contrast, fish in the CR groups showed no changes in CAT activity. Due to the limited available tissue, only select parameters were examined.

### 3.4. Immune Parameters

**Figure 4:**
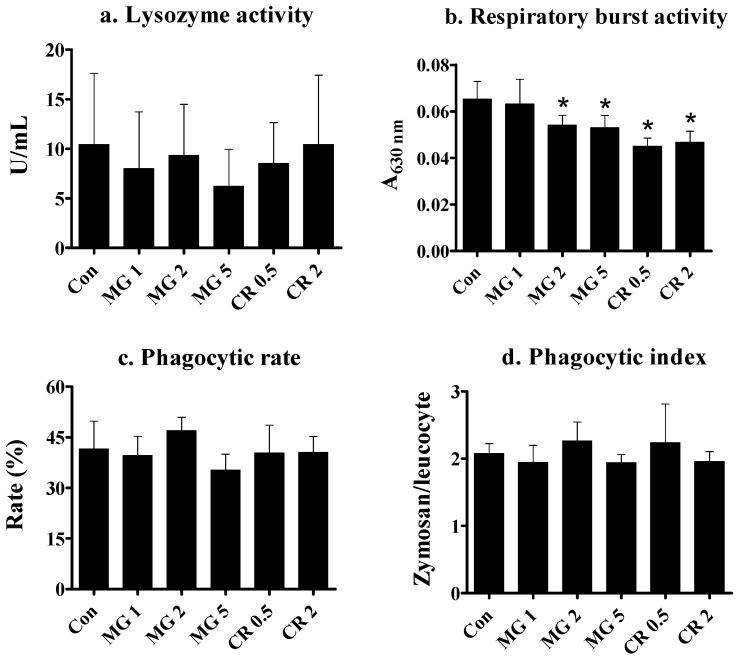
Effects of 5-week MG and CR feeding on direct and indirect innate immune parameters of golden severum. Data are expressed as the mean ± standard deviation from 12 fish per group (4 fish from each of the 3 tanks). * Significant difference from control at *p* < 0.05 with Dunnett’s analysis. Con, control; numbers on the *X*-axis denote diet percentage. (**a**) Lysozyme activity (U/mL): measured by the degradation rate of *Micrococcus lysodeikticus* cell walls; (**b**) Respiratory burst activity (A630 nm): assessed by ROS production using PMA and nitroblue tetrazolium; (**c**) Phagocytic rate (%): percentage of splenic leukocytes engulfing zymosan particles; (**d**) Phagocytic index: average number of zymosan particles engulfed per leukocyte.

To determine whether the two test substances stimulate innate immune functions, several parameters were examined after feeding with test substances. None of the groups fed MG (1, 2, and 5%) or CR (0.5 and 2%) exhibited a statistically significant change in plasma lysozyme activity (Figure 4a).

The respiratory burst activity of spleen leukocytes was estimated by measuring the ROS production rate, which decreased after supplementation with CR at 0.5 and 2.0% or 2–5% MG (Figure 4b).

The phagocytic activity of fish leukocytes after supplementation with MG and CR was examined based on PR, defined by the proportion of leukocytes that engulfed zymosan and PI, defined by the number of zymosan particles engulfed by each leukocyte. No differences in phagocytic activity were observed between the MG and CR groups (Figure 4c,d).

## 4. Discussion

In this study, the effects of lutein-containing MG and CTX-containing CR on the growth, body color development, antioxidant properties, and innate immune capacity of golden severum were examined. Few variations exist in the constituents of different marigold varieties which are not considered here for brevity.

The hypothesis for testing parameters was based on natural substances reported in other animals that contain carotenoids with coloring and antioxidant properties [28]. Furthermore, the anti-oxidative capacity is often linked to innate immune functions since there is a pharmacological crossroad due to the effects of antioxidants on ROS production [29]. Activated immune cells produce ROS as a protective measure against invading pathogens, which can also damage immune cells by oxidizing the polyunsaturated fatty acids in the cell membrane. Endogenous and exogenous antioxidants secure the integrity of immune functions by preventing ROS-induced immune cell damage [30]. Furthermore, irrespective of this correlation, carotenoids have been reported to stimulate immune functions [31].

Feeding diets containing up to 5.0% MG for five weeks did not influence the growth parameters of golden severum in the present study. Hence, the tested concentrations were not overtly toxic. The absence of significant differences in growth parameters suggests that the tested concentrations of MG and CR were within a safe range for use in ornamental fish diets. Similarly, in a previous study, the inclusion of up to 1.6% MG had no effect on the growth of rainbow trout (*Oncorhynchus mykiss*); however, growth inhibition was observed at MG concentrations > 2.4% [10]. The authors attributed their findings to the crude fiber content unintentionally included in the MG diet. It is unclear whether there is a true difference in results between golden severum and rainbow trout, as the exact constituents in the two test cases have not been defined. In rats, feeding 100 mg/kg/day MG for two months did not affect growth [32]. The MG components vary depending on plant origin and extraction procedure; such differences are not only capable of affecting pharmacological efficacy, but they may also cause adverse effects [33]. In the current study, feeding up to 2.0% CR did not influence the body weight of golden severum. Similar results were also observed in rainbow trout, where the fish were fed a diet containing 0.01% CR [34,35]. Although body weight gain is typically considered less important in ornamental fish than in food fish, the overall effects of MG and CR on body weight changes suggest that these two substances are not likely detrimental to general health maintenance within the tested concentration ranges. Meanwhile, the levels set for the current testing, 0.5–5.0%, might be too expensive for feeding food fish.

Body color changes were assessed after feeding either MG or CR, and both increased the lightness values at lower concentrations (MG 1.0% and CR 0.5%). Lightness values did not differ from the control’s when the concentrations were further increased (MG 2–5% and CR 2.0%). These results suggest that carotenoids in MG and CR interact differently with the chromatophores of golden severum, leading to distinct changes in pigmentation depending on concentration. This biphasic effect may have been observed because increasing a particular tone can lead to a decrease in lightness. For example, increasing the tone from yellow to gold-brown by MG or from pink to dark red by CR can reduce the lightness value. The body lightness value of fish is influenced in a complex manner by hormones, background colors, illumination intensity, and dietary constituents [36,37,38,39,40]. Based on the current data, it is challenging to delineate the factors that primarily contributed to the increased lightness.

It was observed that while MG increased the yellow–blue tone, CR increased the red–green tone at all inclusion levels. CTX, the major component of CR, appears red, leading to red enhancement in the muscle of rainbow trout [41]. Moreover, the supply of pure lutein, the main pharmacological substance in MG, or CTX, can intensify the body color of rainbow trout, masu salmon, Japanese eels, dark-banded rockfish, and Korean rockfish [42]. Body color enhancement in aquatic animals by supplementation with MG has not been examined, although a similar activity can be expected, deduced from the reported color improvement effects in poultry eggs [43]. The observed changes in chromaticity may directly reflect the accumulation of carotenoids in the body of golden severum. Furthermore, the present data confirm that optical chromaticity measurements identified subtle differences in color augmentation even when visual determinations could not readily identify subtle differences in the co-existence of other compounding factors.

To evaluate the beneficial pharmacological effects, the antioxidant and innate immune activities were examined after supplementation with either MG or CR. Although the two pharmacological activities are discrete and generally independent, a crossroad biochemical process exists between them. For example, antioxidant capacity helps fight cellular damage caused by free radicals originating from various causes, including pathogen infections [44]. As infections trigger the accelerated production of ROS to remove pathogens, antioxidant molecules can scavenge ROS, leading to reduced pathogen removal. Simultaneously, numerous antioxidant molecules can increase the body’s resistance to infectious pathogens through various mechanisms [45]. Both antioxidant and immunostimulatory activities have consistently been reported for numerous carotenoids [46].

In this study, a tendency for elevated hepatic SOD activity was identifiable with MG administration at medium levels (2%) but not with CR administration at any level. Superoxide dismutase functions as an efficient antioxidant enzyme by scavenging superoxide ions (O_2_^−^) via hydrogen peroxide and molecular oxygen production [47,48]. The SOD-stimulating effect of MG has been reported in terrestrial animals and human cells [19,49,50,51,52]; it appears that a similar mechanism operates in golden severum.

Although CTX has already been used in aquatic animals for body color improvement [53], its role in SOD activity in aquatic species is complex. In black tiger shrimp (*Penaeus monodon*), hepatic SOD activity diminishes after CTX feeding [54]. Interestingly, serum SOD activity was reduced after feeding astaxanthin (a CTX analog), β-carotene, or combined astaxanthin–carotene to the ornamental fish characin (*Hyphessobrycon callistus*) [55]. Although the correlation between hepatic and serum SOD enzyme activities remains unclear, Wang et al. [55] deduced that such a decrease in activity in the serum indicates an elevated antioxidant status in the liver. In contrast, increased hepatic SOD activity following CTX treatment has been reported in mammals [49,56]. It is therefore necessary to determine whether a differential change in enzyme activity occurs between the serum and liver of golden severum. Furthermore, CTX is much weaker than astaxanthin or β-carotene in exerting antioxidant activity, despite their similar chemical structures [57]. Astaxanthin is reportedly ten times more potent than CTX as an antioxidant [58].

GST conjugates electrophilic chemicals to water-soluble substances for their efficient excretion from the body. Thus, GST indirectly contributes to the antioxidant capacity of the cells [59]. Under oxidative stress, GST is rapidly expressed to confer stress tolerance [60,61]. In the present examination of GST after MG and CR administration, no statistically significant changes were observed. It has been reported that CTX does not increase the activity of GST or glucuronosyl transferase (another conjugating enzyme) in the liver of rainbow trout [35,62]. Similarly, MG failed to stimulate GST activity in rats [63] and mice [51]. Thus, the findings in gold severum align with the observations in rainbow trout [35], where GST activity was not influenced by CTX. This contradicts the observations in studies on mice [49] and rats [64], where elevated activity was observed in both species after CTX feeding. As various factors, such as feeding levels and duration, in addition to animal species, can be related to GST activity, further analyses are warranted to understand this discrepancy.

The current results suggest that MG exerts antioxidant activity by directly increasing the activities of SOD and catalase. Since this was only observed up to 2% MG, it can be deduced that there is an optimal level, and excess is not necessarily beneficial. Hence, the active component(s) in MG may have exerted their activity, as various carotenoids have antioxidant potential [65,66].

Tissue TBARS is an indicator of the antioxidant status of an organism, as higher TBARS levels are observed when the antioxidant potential is compromised [67]. Hepatic TBARS levels were significantly lower in golden severum fed MG or CR. These results agree with previous reports, where both MG [51,68] and CTX [49,69] reduced TBARS in terrestrial animals. The observations that TBARS levels were reduced by MG and CR, and the two anti-oxidative enzymes SOD and CAT were elevated only by MA, suggest that CR may have direct antioxidant activity. In contrast, MG appears to have accomplished this activity by activating antioxidant-associated enzymes.

Although the immunostimulatory activity of carotenoids is well known [31,70], there have been a few conflicting reports. For example, Amar et al. [71] observed that CR elevated phagocytic activity and resistance to infectious viruses in rainbow trout. Similarly, the immunomodulatory effects of astaxanthin supplementation were reported in the same fish species [72]. Marigold extract failed to alter complement activity in goldfish [73]. Similarly, supplementation with lutein and CTX did not impact antibody production in hens [74]. The potential of MG as an immune stimulant has been reported in several animals, including rainbow trout, but no similar studies have been conducted in golden severum. Collectively, the results of this study indicate that neither MG nor CR stimulated innate immune functions in golden severum. All direct and indirect parameters for innate immune functions, that is, lysozyme activity and phagocytic activity, were not altered by feed supplementation. Respiratory burst activity was the only parameter that decreased after feeding. From the point of view of innate immunity, this decrease can be viewed as compromised immunity. An elevated antioxidant capacity was identified in the hepatic tissue, whereas diminished respiratory burst activity was observed in isolated leukocytes. This suggests that the production of ROS was diminished by supplementation with either MG or CR. Thus, the reduced respiratory burst activity may reflect reduced ROS levels achieved by antioxidant enzymes at the phagocyte level [44]. Interestingly, elevated ROS levels can be beneficial or detrimental to the host organism, depending on its production level [75]. Generally, slightly elevated levels are beneficial in accomplishing immune functions [76]. Therefore, it is reasonable that the reduced respiratory burst activity indicates the ROS-scavenging role of the test substances.

Finally, this study is limited by the absence of a detailed analysis of the bioactive components in MG. While lutein is identified as the primary active ingredient, the potential influence of other compounds cannot be excluded. Future studies should aim to profile these components and elucidate their individual and combined effects on pigmentation and oxidative stress.

## 5. Conclusions

The results of this study collectively suggest that lutein-containing MG and CTX-containing CR possess distinct body-coloring effects, reflecting the chromogenic properties of the included substances. Optimal pigmentation effects were observed at 1.0% MG, which enhanced the yellow–blue tone, and at 0.5% CR, which intensified the red–green tone. Both supplements increased antioxidant activity, with MG showing enhanced enzymatic activity at 2.0% and CR primarily reducing TBARS levels. However, neither appeared to stimulate non-specific immune functions, as evidenced by the decreased respiratory burst activity. These findings indicate that MG and CR can enhance ornamental value and antioxidant capacity without compromising health under the tested conditions.

## Figures and Tables

**Table 1:** Effects of 5-week MG and CR feeding on general golden severum growth parameters.

Experimental Group						
Parameter	Control	MG 1.0%	MG 2.0%	MG 5.0%	CR 0.5%	CR 2.0%
Survival rate (%)	100	100	98	100	100	97 ^1^
Initial body weight (g)	9.12 ± 0.32	9.16 ± 0.36	9.31 ± 0.57	8.98 ± 0.32	8.99 ± 0.22	8.94 ± 0.41
Final body weight (g)	22.02 ± 0.78	20.20 ± 3.13	18.76 ± 1.40	23.42 ± 0.42	21.39 ± 1.91	20.59 ± 2.65
Weight gain (%)	141.30 ± 0.07	120.70 ± 35.46	102.70 ± 28.27	160.90 ± 9.77	137.70 ± 18.43	131.50 ± 39.25
Specific growth rate (%/day)	2.52 ± 0.00	2.24 ± 0.44	2.00 ± 0.39	2.74 ± 0.11	2.47 ± 0.22	2.37 ± 0.51

Data are presented as the mean ± standard deviation of values from the tank mean (*n* = 3) of each group. As an independent experimental unit, the tank mean (20 fish per tank) was used for analysis. No statistically significant differences were observed between the groups (*p* > 0.05) based on one-way ANOVA. ^1^ Unusual mortality occurred due to fish escaping tanks.

## Data Availability

Dataset available on request from the authors.
